# Perinatal mental health around the world: priorities for research and service development in Italy

**DOI:** 10.1192/bji.2019.31

**Published:** 2020-02

**Authors:** Pietro Grussu, Ilaria Lega, Rosa Maria Quatraro, Serena Donati

**Affiliations:** 1 Family Service Unit, South Padua District, AULSS 6 Euganea, Regione Veneto, National Health Service, Italy. Email: pgruss@tin.it; 2 National Centre for Disease Prevention and Health Promotion, Istituto Superiore di Sanità – Italian National Institute of Heath, Italy; 3 Hospital Psychology, Obstetrics and Gynaecology Unit, AULSS 8 Berica, Regione Veneto, National Health Service, Italy

**Keywords:** Community mental health teams, depressive disorders, perinatal psychiatry

## Abstract

In Italy, most studies on perinatal mental health and initiatives aimed at improving the early detection and management of perinatal mental disorders have been carried out at the local level. National population-based studies are lacking. A study of pregnant women, recruited and diagnosed by a university hospital, found a 12.4% prevalence of minor and major depression during pregnancy, and a prevalence of 9.6% in the postpartum period. In a population-based surveillance system, covering 77% of national births, suicide was identified to be one of the main causes of maternal death within the first year after birth, yet half of those who were known to have a high suicide risk during the postpartum period had not been referred to a mental health service. The value of recognising depressive or anxiety symptoms early, during pregnancy, has been emphasised by recent research and should be linked to multi-professional psychosocial interventions. Since 2017, the Italian public primary care services that are dedicated to pregnancy assistance (Family Care Centres) have been tasked to provide free psychological assessment to pregnant and postpartum women. Action is now needed in order to improve access to Italian Family Care Centres for pregnant women and to develop an integrated care model involving obstetric and mental health services.

Italy had about 60.3 million inhabitants and more than 430 000 live births per year in 2018. Free comprehensive coverage is provided by the National Health Service (NHS). Responsibility for healthcare is shared by the central government and the 20 Italian regions. All citizens have access to healthcare coverage through local health units (LHUs), each of which manages a geographically defined catchment area. Treatment in public and accredited private hospitals is free of charge to the whole country.

After many years of limited interest in perinatal psychopathology, there has been growing awareness of the problem, owing to several events. First, in the early 2000s, the Edinburgh Postnatal Depression Scale (EPDS) was available in Italian translation.^1^ Second, a research group of experts on postpartum depression was established, coordinated by Piero Morosini at the Italian National Institute of Health. Third, the document *Help to Pregnant Women and Postpartum Depression* was issued by the Italian National Bioethics Committee in 2005.^2^ These events stimulated research and training on the topic of perinatal mental health.

A growing body of initiatives aimed at fostering research into perinatal mental disorders, including prevention and treatment, have been developed over the past 20 years. Research in Italy on perinatal mental health and implemented care models for treatment has mostly been carried out at a local level, and consequently has limited generalisability. Nevertheless, the limited evidence that is available highlights unmet health needs among Italian women, during or after pregnancy, many of whom have symptoms or a diagnosis of mental disorder, and emphasises the need for action.

## Perinatal mental health research in Italy

National-level and population-based studies on perinatal mental disorders are lacking, but local studies have pointed to the magnitude of the problem. In a sample of 1066 women who were assessed by the Perinatal Depression-Research and Screening Unit study and Department of Psychiatry and Department of Obstetrics and Gynaecology of the Azienda Ospedaliero Universitaria Pisana^3^ with the EPDS and the Structured Clinical Interview for DSM-IV Axis I (SCID-I), the period prevalence of minor and major depression was found to be 12.4% during pregnancy and 9.6% in the postpartum period; 1.6% of women had a new episode of depression during pregnancy, and 5.7% in the postpartum period.^3^ Suicidality was assessed in the same sample by self-report, using the EPDS. This showed a period prevalence of 12% during pregnancy and 8.6% during the postpartum period.^4^ Among women with a SCID-I diagnosis of major or minor depressive episode, the period prevalence of self-reported suicidality was much higher, at 30.6%.^4^

The Italian Obstetric Surveillance System recognises suicide as among the main causes of maternal death.^5^ Over the period from 2006 to 2012, in ten Italian regions covering 77% of total national births, 567 cases of maternal suicide were recorded during pregnancy or within 1 year after birth, induced abortion or miscarriage. Most women (60%) who died by suicide had a psychiatric history, with bipolar disorder and major depressive disorder being the most frequent prior diagnoses. Half of those women who were known to be at high suicide risk during the postpartum period were not subsequently seen by a secondary mental health service.^6^

The main risk factors for postpartum depression that have been identified by Italian studies are similar to those reported in the international literature. A study of three LHUs recruited a sample of 2668 pregnant women, most of whom were attending antenatal classes.^7^ Symptoms of depression and anxiety, and a lack of psychological support from family and friends during pregnancy, were associated with high levels of depression symptoms postpartum. In another study, 389 women were contacted 8 months after giving birth in a tertiary-level hospital in Trieste. A questionnaire survey found that 10% were experiencing violence from their partner or another family member.^8^ Depressive symptoms were much more common in this subset, with an odds ratio of >19. It is not known whether these risk factors are specific to Italian subjects, owing to methodological differences and the different assessment tools used by researchers.^9^

Recent Italian studies on the value and effectiveness of psychosocial interventions for women who have perinatal affective disorders or are at risk of postpartum depression reflect a growing interest in the field. Cauli and colleagues^10^ recruited a sample of 318 women with prenatal anxiety or depressive symptoms during pregnancy from a university hospital in northern Italy. After a multidisciplinary psychosocial intervention (MPI), they found some positive effects in terms of postpartum recovery, symptom reduction and secondary prevention of further depression. The MPI involved prenatal screening for depression and anxiety. Women who screened positive for mental disorders were offered different intervention options. These included interpersonal psychotherapy sessions, pharmacological treatment and psychosocial counselling. The choice of intervention depended on symptoms' severity and the women's individual preferences. Steardo and colleagues^11^ recently started a randomised controlled trial aimed at evaluating the efficacy and effectiveness of a psychoeducational family intervention in improving depressive symptoms among patients with perinatal depression.

## Perinatal mental health service in Italy

According to Italian national health policies, maternal care is universally accessible. Maternal care services are provided free of charge within the public sector. Family Care Centres (FCCs) are the Italian NHS's primary care services dedicated to pregnancy assistance. Formally established in 1975, there are now more than 1800 national FCCs. Their approach is based on a multidisciplinary, proactive and holistic approach. They should ensure that both pre- and postnatal assistance are provided to mothers and their families, including maternal and fetal assessments, antenatal classes, breastfeeding promotion and postpartum support.

Since 2017, the Italian Ministry of Health has directed that psychological assessments are to be included in the care provided by FCCs to women during pregnancy and after birth, for prevention and early recognition of perinatal mental disorders. This directive established for the first time a clinical awareness that the care provided to pregnant and postpartum women in the Italian NHS should encompass attention to their mental health. A corollary of this directive is that there should be prompt availability of specialised consulting and care for women with identified perinatal mental health needs.

There are no NHS perinatal out-patient or in-patient mental health services in Italy, and only a few university psychiatric clinics have established specific care pathways for perinatal women with mental disorders. In general, mental healthcare is delivered by a network of mental health departments. Community mental health centres (CMHCs) provide adult psychiatry services in out-patient settings, including consulting and coordinated care with primary care services. Despite being highly accessible and free of charge, CMHCs are often located separately from primary care services. Consequently, they are often perceived as being facilities for severely mentally ill people. Fewer than 40% of patients with symptoms of depression that have been recognised by primary care services were referred on to secondary psychiatric care in an Italian urban setting.^12^ Recent findings on the risk of maternal suicides highlights the lack of continuity of care between primary, mental health and maternity care services. This problem is not peculiar to Italy and exists in many other European countries.^6^

In 2018, the Ministry of Health supported a pilot study which implemented postpartum depression management initiatives in 16 Italian regions. The first results of these regional experiences will hopefully help to identify effective models for mental health promotion, prevention and intervention in the perinatal period.

## Discussion

The limited Italian evidence on perinatal mental health indicates the need for research involving a nationally representative sample of pregnant and postpartum women and their partners. As well as epidemiological investigations, which could be used to estimate the prevalence of perinatal psychopathologies within the country, further studies are required to assess the applicability of effective interventions. Italy should learn from other countries' experiences, in order to more effectively support and treat women affected by perinatal mental illness. Research priorities in Italy include gathering better evidence on the accessibility of perinatal services, reducing the stigma that accompanies perinatal mental disorders, evaluating appropriate early detection tools and treatment models, and clarifying the role of peer support and the economic costs of untreated perinatal psychopathology.

From 2017, FCCs have been the hub of the perinatal mental healthcare pathway in Italy. Multidisciplinary primary care services devoted to the care of women and their families are undoubtedly the best place to anchor the renewed national commitment to promote perinatal mental health. The Italian NHS is not used by everyone, and several areas of the country have high proportions of patients receiving private antenatal care. Action is needed to improve the accessibility of FCCs to all pregnant women and to develop an integrated care model between FCCs, maternity wards and secondary-level mental health services.
