# Perinatal mental health around the world: priorities for research and service development in Italy – CORRIGENDUM

**DOI:** 10.1192/bji.2020.10

**Published:** 2022-08

**Authors:** Pietro Grussu, Ilaria Lega, Rosa Maria Quatraro, Serena Donati

**Keywords:** Community mental health teams, depressive disorders, perinatal psychiatry, corrigendum

This article was published with an error on p. 9, which states that over the period from 2006 to 2012, in ten Italian regions covering 77% of total national births, 567 cases of maternal suicide were recorded during pregnancy or within 1 year after birth, induced abortion or miscarriage. In fact there were only 67 cases of maternal suicide.
